# A retrospective study using machine learning to develop predictive model to identify urinary infection stones in vivo

**DOI:** 10.1007/s00240-023-01457-z

**Published:** 2023-05-31

**Authors:** Yukun Wu, Qishan Mo, Yun Xie, Junlong Zhang, Shuangjian Jiang, Jianfeng Guan, Canhui Qu, Rongpei Wu, Chengqiang Mo

**Affiliations:** 1 Department of Urology, The First Affiliated Hospital of Sun Yat-sen University, No. 58, Zhongshan 2nd Road, Guangzhou, 510080 Guangdong China; 2 Department of Urology, Guangzhou Panyu Central Hospital, Guangzhou, 510080 Guangdong China

**Keywords:** Infection stones, Machine learning, Prediction model, Struvite, Urolithiasis

## Abstract

Preoperative diagnosis of urinary infection stones is difficult, and accurate detection of stone composition can only be performed ex vivo. To provide guidance for better perioperative management and postoperative prevention of infection stones, we developed a machine learning model for preoperative identification of infection stones in vivo. The clinical data of patients with urolithiasis who underwent surgery in our hospital from January 2011 to December 2015 and January 2017 to December 2021 were retrospectively analyzed. A total of 2565 patients were included in the study, and 1168 eligible patients with urinary calculi were randomly divided into training set (70%) and test set (30%). Five machine learning algorithms (Support Vector Machine (SVM), Multilayer Perceptron (MLP), Decision Tree (DT), Random Forest Classifier (RFC), and Adaptive Boost (AdaBoost)) and 14 preoperative variables were used to construct the prediction model. The performance measure was the area under the receiver operating characteristic curve (AUC) of the validation set. The importance of 14 features in each prediction model for predicting infection stones was analyzed. A total of 89 patients (5.34%) with infection stones were included in the validation set. All the five prediction models showed strong discrimination in the validation set (AUC: 0.689–0.772). AdaBoost model was selected as the final model (AUC: 0.772(95% confidence interval, 0.657–0.887); Sensitivity: 0.522; Specificity: 0.902), UC positivity, and urine pH value were two important predictors of infection stones. We developed a predictive model through machine learning that can quickly identify infection stones in vivo with good predictive performance. It can be used for risk assessment and decision support of infection stones, optimize the disease management of urinary calculi and improve the prognosis of patients.

**Supplementary Information:**

The online version contains supplementary material available at 10.1007/s00240-023-01457-z.

## Introduction

Urolithiasis is a relatively common disease in urology, and its prevalence has been increasing worldwide over the past few decades [1, 2]. Studies have shown that about 1/17 Chinese adults have kidney stones [3], the recurrence rate was estimated to be 67% within 5 years. Infection stones account for 10%–15% of urolithiasis, and is a specific type of urolithiasis associated with urinary tract infection (UTI) caused by urease producing organisms [4]. It can rapidly grow into giant staghorn stones within 4 to 6 weeks, and struvite is generally considered to be an independent risk predictor for infectious-related complications, such as sepsis, in patients after percutaneous nephrolithotomy [5, 6]. Patients with infection stones represent one of the most challenging populations of patients with urolithiasis due to their complex structure and high recurrence rate [7, 8]. Stone composition is the basis for further diagnosis and treatment decisions, and the management of infection stones should start with early and correct identification [9, 10]. At present, there are some predictive models to distinguish infection stones from non-infection stones. However, there are few reports on preoperative prediction models that can achieve rapid, simple, and in vivo prediction based on large samples.

The development of machine learning algorithms may provide an opportunity for early preoperative prediction of infection stones by integrating large amounts of data such as demographics, diagnostics, routinely collected measurements, and interventions [11]. It can effectively deal with the nonlinear relationship and high-dimensional space in medical data, with high accuracy and good generalization in the field of urinary calculi, which outperform traditional modeling methods [12]. Machine learning has been applied in biomedical fields such as disease diagnosis, outcome prediction, medical image analysis, and therapeutics [13, 14]. Therefore, in this study, we sought to develop machine learning models that can be used to differentiate infection and non-infection stones before necessary surgery is performed on patients with urinary stones to better guide perioperative management and prevent the occurrence of infection stones after surgery.

## Material and methods

### Patients

The study was approved by the ethics committee of the First Affiliated Hospital of Sun Yat-sen University, and the requirement for informed consent was waived (No.: IIT-2022-437). The clinical data of 2565 patients who underwent surgery for urinary calculi in our hospital from January 2011 to December 2015 and January 2017 to December 2021 were retrospectively analyzed (the test was not performed in 2016 due to mechanical reasons). After excluding patients with incomplete clinical data, 1168 patients were used for modeling. Baseline clinical data were obtained from medical records, including age, sex, urinalysis and urine culture, a total of 24 indicators. The composition of the stones was analyzed by Fourier transform infrared spectroscopy, and the main stone components were recorded. The stone component with the highest proportion was selected as the main stone component. When magnesium ammonium phosphate hexahydrate occurs, the main ingredient is determined to be magnesium ammonium phosphate hexahydrate, regardless of the proportion. Infection stones mainly include magnesium ammonium phosphate hexahydrate and calcium carbonate stones. Others were considered to be non-infection stones.

### Model

The samples were randomly divided into a training set and a validation set at a ratio of 7∶3 for the establishment and validation of the model, respectively. Five machine learning algorithms including SVM, MLP, DT, RFC, and AdaBoost were used to establish the prediction model. SVM solves the binary classification problem by fitting a maximum margin discriminator to a dataset in a kernel-induced feature space, and it has been applied in many medical diagnostics and disease classifications [15]. The MLP architecture consists of multiple interconnected hidden neurons, and the PyTorch framework is used to build and train the MLP model. We performed a semi-systematic grid search to explore the models that could be generated using multiple different combinations of the presented hyperparameters [16]. In the DT, the root node of the tree will be the feature that optimally partitions the training data. The threshold that maximizes the homogeneity of the sample subgroups is found by repeating this step [17]. RFC is a tree-based algorithm that integrates multiple decision trees by majority voting to determine the classification result [18]. Applying the boosting algorithm AdaBoost [19] provides a correction mechanism to improve the model after each prediction of the patient state [20]. Ultimately, the decision is the result of the summation of all the basic models. It is one of the most effective techniques in machine learning.

### Data analysis

SPSS 26.0 software was used to analyze the data. Measurement data were expressed as mean ± standard deviation (SD), the *t*-test was used for normal distribution and the Mann–Whitney U test was used for non-normal distribution. The Chi-square test or Fisher exact test was used to compare the differences between the two groups. Statistical significance was defined as two-sided *P* < 0.05. Logistic regression was used for univariate regression analysis, and the factors with higher degrees of freedom were selected to construct the prediction model. receiver operating characteristic (ROC) and area under the curve (AUC) was used to evaluate the ability of each model to distinguish non-infectious and infectious stones. The 95% confidence interval (CI) of AUC and the difference in AUC values among different models were tested to determine the best threshold of infection stones Sensitivity, specificity, and accuracy were calculated at the optimal threshold.

## Results

### Patients

Table 1 presents the clinical data from the demographic, stone composition analysis based on the gender of 2565 patients. The average age of the patients was 52.14 years old, with 65.07% of males and 34.93% of females. The highest incidence of stones in males was 41–50 years old (25.04%), and that in females was 51–60 years old (33.82%). In terms of stone composition, there were 1770 cases (69.01%) of calcium oxalate stones, 482 cases (18.79%) of uric acid stones, 118 cases (4.6%) of calcium phosphate stones, and 189 cases (7.37%) of infection stones. The proportion of infection stones in men was lower than that in women (M/F = 0.64, P < 0.001). The spectrum of pathogens isolated from urine cultures is shown in Supplementary Figure S1. The most common pathogen of non-infection stones was Escherichia coli (107 strains), followed by Enterococcus faecalis (20 strains) and streptococcus agalactiae (14 strains). proteus mirabilis was the most common pathogen of infection stones (18 strains), followed by Escherichia coli (11 strains) and klebsiella pneumoniae (7 strains). Supplementary Figure S2 shows the urine pH level distribution of infection stones and non-infection stones. Among the infection stones, 44.94% of the patients had a urine pH of 6.0, 17.98% had a urine pH of 6.5, and 25.84% had a urine pH of 7.0. Among the non-infection stones, 11.39% of the patients had a urine pH of 5.0, 50.28% had a urine pH of 6.0 and 25.28% had a urine pH of 6.5. In terms of timeline, the incidence of urolithiasis increased, and the ratio of males to females decreased, but it did not reach statistical significance. The incidence of infection stones increased, and the incidence of uric acid stones decreased, indicating that the health management of uric acid stones had improved (Table 2). A total of 35 patients had at least second recurrence, of which 34.3% had inconsistent recurrence components, and the incidence of infection stones was increasing (5 cases) (Table 3).

**Table 1:** Characteristics of patients with urolithiasis according to the gender

Characteristics	Overall	Male	Female	Ratio (M/F)	*P *value
Number of cases	2565	1669	896	1.86	/
Age, years, n (%)					
Mean, years	52.14	51.92	52.56	0.99	/
18–30	166 (6.47%)	107 (6.41%)	59 (6.58%)	1.81	/
31–40	380 (14.81%)	273 (16.36%)	107 (11.94%)	2.55	/
41–50	590 (23.00%)	418 (25.04%)	172 (19.20%)	2.43	/
51–60	700 (27.29%)	397 (23.79%)	303 (33.82%)	1.31	/
61–70	491 (19.14%)	296 (17.74%)	195 (21.76%)	1.52	/
≥ 71	238 (9.28%)	178 (10.67%)	60 (6.70%)	2.97	/
Infection stones, n (%)	189 (7.37%)	74 (4.43%)	115 (12.83%)	0.64	0.000
Struvite	152 (5.93%)	59 (3.54%)	93 (10.38%)	0.63	0.000
Carbapatite	37 (1.44%)	15 (0.90%)	22 (2.46%)	0.68	0.002
Non-infection stones, n (%)	2376 (92.63%)	1595 (95.57%)	781 (87.17%)	2.04	0.000
Calcium oxalate	1770 (69.01%)	1175 (70.40%)	595 (66.41%)	1.97	0.000
Urate	482 (18.79%)	348 (20.85%)	134 (14.96%)	2.60	0.000
Calcium phosphate	118 (4.60%)	69 (4.13%)	49 (5.47%)	1.41	0.124
Cystine	6 (0.23%)	3 (0.18%)	3 (0.33%)	1.00	0.438

**Table 2:** Characteristics of patients with urolithiasis according to the timeline

Characteristics	2011–2015	2017–2021	*P *value
	n = 491	n = 2074	
Gender (%)			
Male, n (%)	324 (65.99%)	1345 (64.85%)	0.635
Female, n (%)	167 (34.01%)	729 (35.15%)	0.635
Ratio (M/F)	1.94	1.84	/
Age, years	49.57	52.75	/
Male, mean	50.05	52.37	/
Female, mean	48.64	53.46	/
Infection stones, n (%)	22 (4.48%)	167 (8.05%)	0.006
Struvite	19 (3.87%)	133 (6.41%)	0.032
Carbapatite	3 (0.61%)	34 (1.64%)	0.086
Non-infection stones, n (%)	469 (95.5%)	1907 (91.95%)	0.006
Calcium oxalate	323 (65.6%)	1447 (69.77%)	0.086
Urate	122 (25.1%)	360 (17.36%)	0.000
Calcium phosphate	21 (4.3%)	97 (4.68%)	0.704
Cystine	3 (0.6%)	3 (0.14%)	0.054

**Table 3:** The distribution of the main urinary stone constituents in patients with urolithiasis recurrence

Characteristics (n = 35)	1st occurrence of urolithiasis	2nd occurrence of urolithiasis	*P* value
Same composition, n (%)	23 (65.7%)		/
Different composition, n (%)	12 (34.3%)		/
Infection stones, n (%)	0	5 (14.3%)	0.020
Struvite	0	3 (8.6%)	0.077
Carbapatite	0	2 (5.7%)	0.151
Non-infection stones, n (%)	35	30	0.020
Calcium oxalate	21 (60.0%)	19 (54.3%)	0.629
Urate	10 (28.6%)	9 (25.7%)	0.788
Calcium phosphate	3 (8.6%)	2 (5.7%)	0.643
Cystine	1 (2.9%)	0	0.314

### Model

**Fig. 1:**
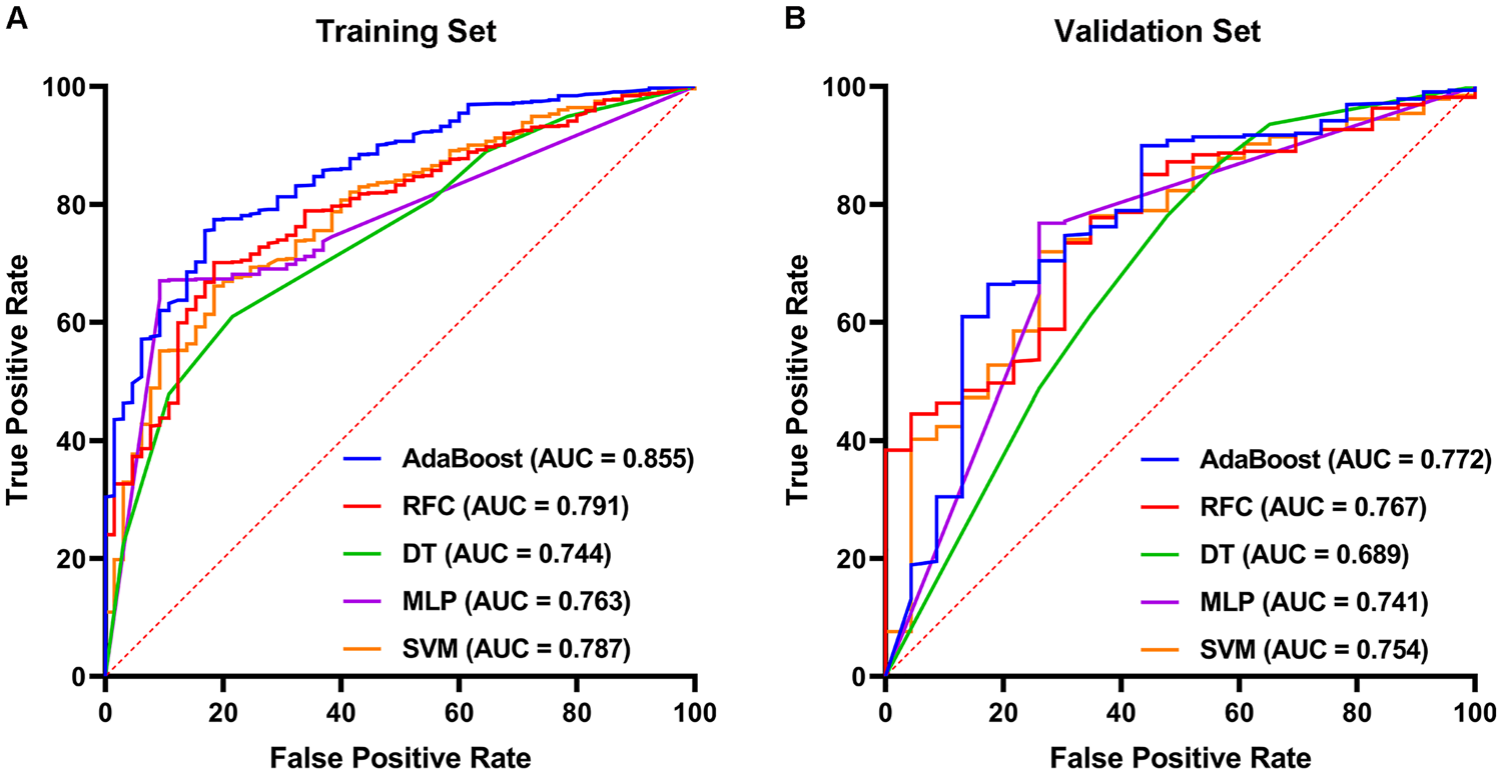
Receiver operating characteristic curves of the machine learning models in the Training Set (**A**) and Validation Set (**B**). The horizontal axis represents False Positive Rate and the vertical axis represents True Positive Rate. AUC closer to 1 indicates better prediction performance. *AdaBoost* adaptive boosting, *RFC* random forest classifier, *DT* decision tree, *MLP* multilayer perceptron, *SVM* support vector machine, *AUC* area under the receiver operating characteristic curve

A total of 1168 patients participated in the modeling, we randomly assigned 70% of the patients to the training set and the remaining 30% to the test set, where infection stones accounted for approximately the same proportion in the training set (7.6%) and the validation set (9.7%), and no significant differences in any variables were found between the training and validation set (Table 4). In the training set, Univariate analysis showed that a total of 14 factors, such as urine culture, urine pH value, and gender, were significantly different between the patients with infection stones and non-infection stones, and the degree of freedom was 1, which was closely related to the occurrence of infection stones (Table 5). Machine learning algorithms were used to construct predictive models from these factors. The AUC, specificity, sensitivity, and accuracy of each model in the training and validation set are shown in Supplementary Table S1 and Table 6, respectively. The receiver operating characteristic curves of the different models are shown in Fig. 1A and B. The AUC values of SVM, MLP, DT, RFC, and AdaBoost in the test set were 0.754 (95% CI 0.637–0.872), 0.741 (95% CI 0.622–0.860) and 0.689 (95% CI 0.566–0.813), respectively, 0.767 (95% CI 0.651–0.883), 0.772 (95% CI 0.657–0.887). The sensitivity values of the five machine learning model scores ranged from 0.522 to 0.739, the specificity values ranged from 0.677–0.902, and the accuracy values ranged from 0.681 to 0.877. After considering other scores, especially prediction accuracy, the AdaBoost model was selected as the final prediction model.

**Table 4:** Baseline characteristics of the patients in predicting infection stones

Characteristics	Training set		Validation set	
	Non-infection stones	Infection stones	Non-infection stones	Infection stones
Gender	759	58	320	31
Male (n%)	513 (67.59%)	27 (46.55%)	210 (65.63%)	9 (29.03%)
Female (n%)	246 (32.41%)	31 (53.45%)	110 (34.37%)	22 (70.97%)
Age, year	53.15 ± 13.31	52.91 ± 10.74	52.53 ± 12.75	53.29 ± 13.33
Weight, kilogram	64.98 ± 11.63	61.45 ± 9.21	63.51 ± 11.82	59.68 ± 11.94
Height, centimeter	164.73 ± 7.63	161.24 ± 6.15	163.86 ± 8.09	159.29 ± 6.69
Body mass index	23.85 ± 3.29	23.60 ± 3.04	23.54 ± 3.39	23.48 ± 4.36
Urine pH	6.15 ± 0.59	6.51 ± 0.67	6.17 ± 0.61	6.60 ± 0.62
Urine specific gravity	1.01 ± 0.04	1.01 ± 0.01	1.02 ± 0.01	1.01 ± 0.00
Urine turbidity				
Negative (n%)	204 (26.88%)	32 (55.17%)	248 (77.50%)	12 (38.71%)
Positive (n%)	555 (73.12%)	26 (44.83%)	72 (22.50%)	19 (61.29%)
Urine nitrite				
Negative (n%)	676 (89.06%)	42 (72.41%)	288 (90.00%)	21(67.74%)
Positive (n%)	83 (10.94%)	16 (27.59%)	32 (10.00%)	10 (32.26%)
Urine glucose				
Negative (n%)	728 (95.92%)	55 (94.83%)	304 (95.00%)	30 (96.77%)
Positive (n%)	31 (4.08%)	3 (5.17%)	16 (5.00%)	1 (3.23%)
Urine protein				
Negative (n%)	475 (62.58%)	25 (43.10%)	212 (66.25%)	11 (35.48%)
Positive (n%)	284 (37.42%)	33 (56.90%)	108 (33.75%)	20 (64.52%)
Urine occult blood				
Negative (n%)	158 (20.82%)	5 (8.62%)	56 (17.50%)	2 (6.45%)
Positive (n%)	601 (79.18%)	53 (91.38%)	264 (82.50%)	29 (93.55%)
Urine leukocyte esterase				
Negative (n%)	221 (29.12%)	5 (8.62%)	74 (23.13%)	1 (3.23%)
Positive (n%)	538 (70.88%)	53 (91.38%)	246 (76.87%)	30 (96.77%)
Urine RBC counts	206.27 ± 648.34	137.07 ± 328.06	198.46 ± 819.71	178.16 ± 324.45
Urine WBC counts	180.80 ± 353.29	331.64 ± 472.12	161.89 ± 324.08	377.81 ± 285.73
Squamous epithelial cells	2.11 ± 7.31	3.71 ± 16.64	2.33 ± 10.37	7.94 ± 15.28
Non-squamous epithelial cells	0.58 ± 0.99	0.76 ± 1.23	0.63 ± 1.42	1.29 ± 1.49
Pathologic casts	0.11 ± 0.32	0.13 ± 0.34	0.12 ± 0.43	0.22 ± 0.40
Hyaline casts	0.17 ± 0.44	0.03 ± 0.18	0.16 ± 0.38	0.00 ± 0.00
Crystals	5.22 ± 23.42	2.71 ± 8.58	4.10 ± 12.76	1.00 ± 2.14
Bacteria	140.56 ± 261.61	260.98 ± 379.38	139.74 ± 254.73	203.48 ± 223.02
Mucus threads	134.20 ± 181.69	118.05 ± 147.52	128.79 ± 151.06	106.29 ± 88.33
Urine culture				
Negative (n%)	590 (77.73%)	24 (41.38%)	255 (79.69%)	14 (45.16%)
Positive (n%)	169 (22.27%)	34 (58.62%)	65 (20.31%)	17 (54.84%)
Urease-producing bacteria				
Negative (n%)	728 (95.92%)	39 (67.24%)	307 (95.94%)	21 (67.74%)
Positive (n%)	31 (4.08%)	19 (32.76%)	13 (4.06%)	10 (32.26%)

*RBC* Red blood cell; *WBC* white blood cell; Data are presented as mean ± standard deviation

**Table 5:** Univariate logistic regression analysis for predictors of infection stones

Variables	Score	Degree of freedom	*P* value
Urine culture	57.980	1	0
Urine pH	33.567	1	0
Gender	27.913	1	0
Urine turbidity	27.059	1	0
Height	23.713	1	0
Urine white blood cell counts	21.787	1	0
Urine protein	17.825	1	0
Urine leukocyte esterase	17.776	1	0
Bacteria	12.398	1	0
Squamous epithelial cells	9.829	1	0.002
Urine specific gravity	9.658	1	0.002
Weight	9.355	1	0.002
Non-squamous epithelial cells	7.921	1	0.005
Urine occult blood	7.549	1	0.006

**Table 6:** Summary of AUC, accuracy, sensitivity, specificity of different models in the validation set

	Accuracy	Sensitivity	Specificity	AUC	95% CI
SVM	0.681	0.739	0.677	0.754	(0.637, 0.872)
MLP	0.687	0.739	0.683	0.741	(0.622, 0.860)
DT	0.764	0.522	0.780	0.689	(0.566, 0.813)
RFC	0.732	0.696	0.735	0.767	(0.651, 0.883)
AdaBoost	0.877	0.522	0.902	0.772	(0.657, 0.887)

*SVM* Support vector machine, *MLP*  multilayer perceptron, *DT*  decision tree, *RFC* random forest classifier, *AdaBoost*  adaptive boosting, *AUC*  area under the receiver operating characteristic curve

## Discussion

In this study, we explored the applicability of machine learning methods to distinguish infection stones from non-infection stones preoperatively in patients. Among the five machine learning models, the AdaBoost model had the highest AUC. Due to the complexity of infection stones, clinical models integrating conventional parameters may be more effective predictors than considering any parameter alone. One possible way to achieve this is to utilize advanced machine-learning methods that have been applied to the prevention and management of infection stones. The construction of the prediction model is derived from common clinical parameters, which are simple, easy to perform, and do not require high technical requirements. It is suitable for promotion in primary hospitals, thus expanding the application prospect of this study.

With the progress and development of minimally invasive surgical techniques and endoscopic instruments, traditional open surgery has been gradually replaced by a variety of minimally invasive surgical methods. The determination of stone types can guide the clinical selection of appropriate treatment methods, and provide a basis for the etiological analysis and the formulation of reasonable surgical plans [21]. Infection stones, which are composed of magnesium ammonium phosphate, carbonate apatite, or ammonium urate, are easily crushed, but can also cause systemic infection after lithotripsy. Therefore, surgeons should remove infection stones as much as possible to avoid residual stones during the operation. Effective antimicrobial therapy is an appropriate intervention for patients with urinary tract infections and recurrent stones [22, 23]. Patients with infection stones may have high rates of infectious complications and mortality, with or without treatment [24, 25]. The mean concentration of serum endotoxin in patients with infection stones was 35 times higher than that in patients with non-infection stones [26].

The formation of infection stones is closely related to urease-producing bacteria. In the present study, positive preoperative urine culture was a predictor of infection stones [27, 28]. As long as urease-producing bacteria appear, the possibility of infection stones should be considered first (Table S2). However, the positive rate of urease-producing bacteria culture is not high at present, it may be that the existing culture medium may not be suitable for the growth of urease-producing bacteria. In the future, renal pelvic urine culture or even stone culture may be needed to further increase the positive rate, and direct detection of urinary microbiota may be considered to be closer to reality. When the prediction model consider that the urolithiasis is infection stones, the treatment should be based on the urine culture analysis (Figure S1). Furthermore, when these urease-producing organisms infect the urinary tract, urea is broken down into ammonia and carbon dioxide in the presence of urease [7], thereby raising urine pH and increasing the concentrations of NH_4_^+^, CO_3_^2−^, and PO_4_^3−^. It has been shown that the crystallization of carbonate apatite begins at a pH greater than 6.8, whereas the crystallization of struvite occurs at a pH greater than 7.2, and the higher the urine pH, the higher the probability of infection stones [29]. In fact, an alkaline urine favors the crystallization of stones containing calcium and phosphate [30]. This is to some extent consistent with the results of our study (Figure S2). Interestingly, our study showed that although the urine pH of infection stones was indeed more alkaline than that of non-infection stones, about half of the patients (44.94%) still had a pH of 6.0, for which a more personalized treatment plan is needed.

Meanwhile, our study also found that for patients with recurrences more than once, the composition of recurrent stones was not completely consistent, and the incidence of infection stones increased with recurrence. It is very important to remove the stones thoroughly during the operation, antibiotics should be used in the perioperative period, and the corresponding dietary structure should be adjusted according to the composition of the stones after the operation. The treatment of infection stones, a special subset of urolithiasis formed by urinary tract infection, is particularly challenging [4, 31], which carries a high risk of postoperative infectious complications that may lead to life-threatening conditions such as severe sepsis and septic shock [32]. Although the use of antibiotics before and after surgery is essential for the adjuvant management of infection stones, the duration and mode of antibiotic therapy are not addressed in current clinical guidelines [33]. Urease inhibitors can directly interfere with the growth process of infection stones precursors and are recommended for patients with surgical contraindications or recurrent infections even after the treatment of infection stones. Urease inhibitors alter urine pH to avoid sedimentation and clearance of infected stones [34].

Our study has some limitations. First, this study was a single-institution retrospective study with a limited number of cases and some selection bias, and the lack of multicenter external validation limits the satisfactory generalizability of the model to other cohorts. At present, the prediction performance is not accurate enough, and other urine indicators, such as urine microorganisms and imaging features, need to be further added to improve the prediction performance. Further work should include optimization and external validation of the model in a larger cohort from multiple centers.

## Conclusions

In conclusion, we developed a preoperative prediction model using machine learning to identify urinary infection stones in vivo. The model is easy to use for both clinicians and patients and may allow clinicians to predict stone types more precisely before surgery, to optimize the disease management of urolithiasis and improve the prognosis of patients.

### Supplementary Information

Below is the link to the electronic supplementary material.

Supplementary file1 (TIF 1237 KB)

Supplementary file2 (TIF 668 KB)

Supplementary file3 (DOCX 12 KB)

## Data Availability

The data used to support the findings of this study are included within the article.
