# Supplementary figures and images for: A retrospective study using machine learning to develop predictive model to identify urinary infection stones in vivo
Source: Urolithiasis. 2023 May 31;51(1):84. doi: 10.1007/s00240-023-01457-z (PMC10232574; doi:10.1007/s00240-023-01457-z)

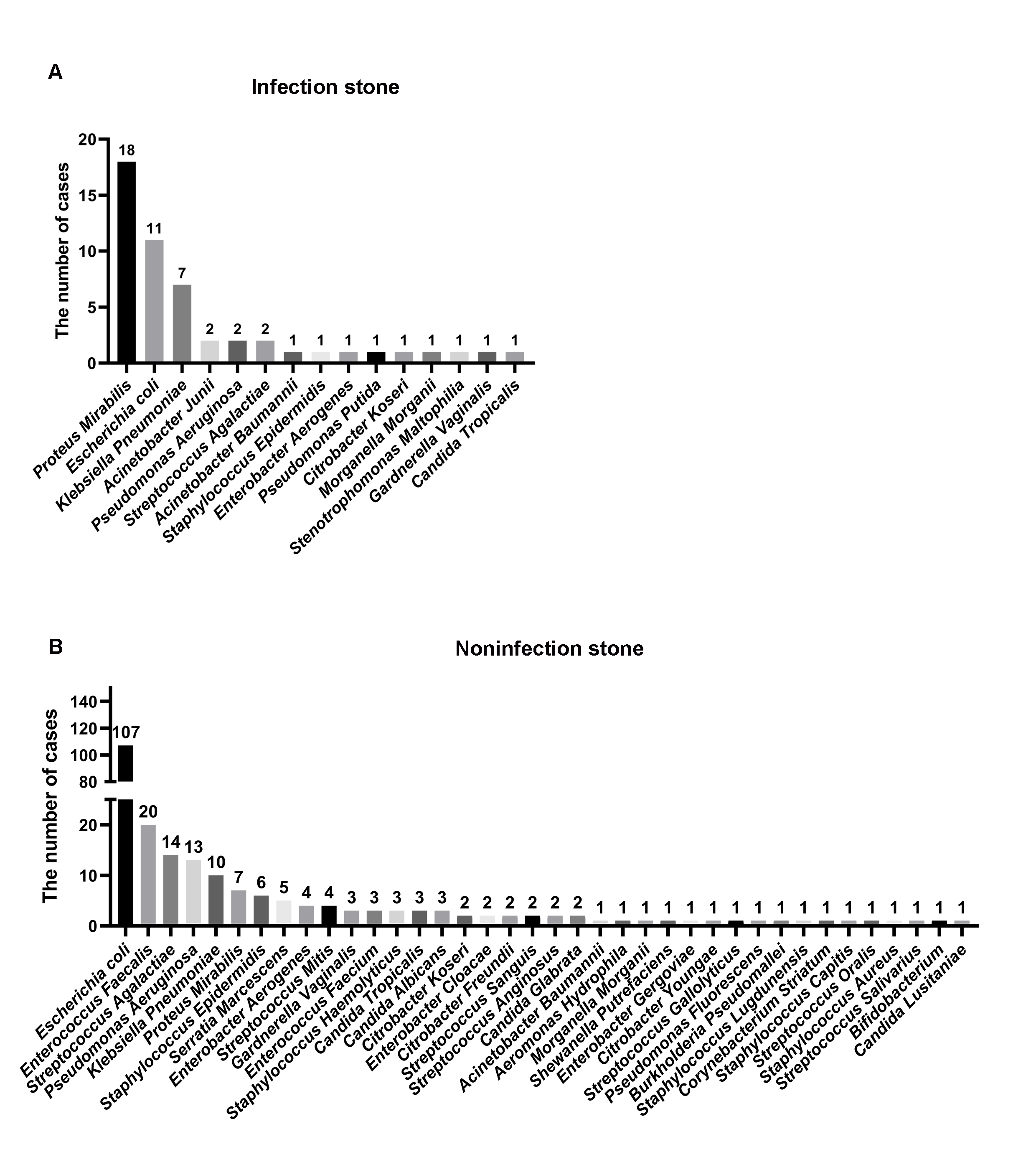

Supplement: Supplementary file 1 — Supplementary file1 (TIF 1237 KB) [file 240_2023_1457_MOESM1_ESM.tif]

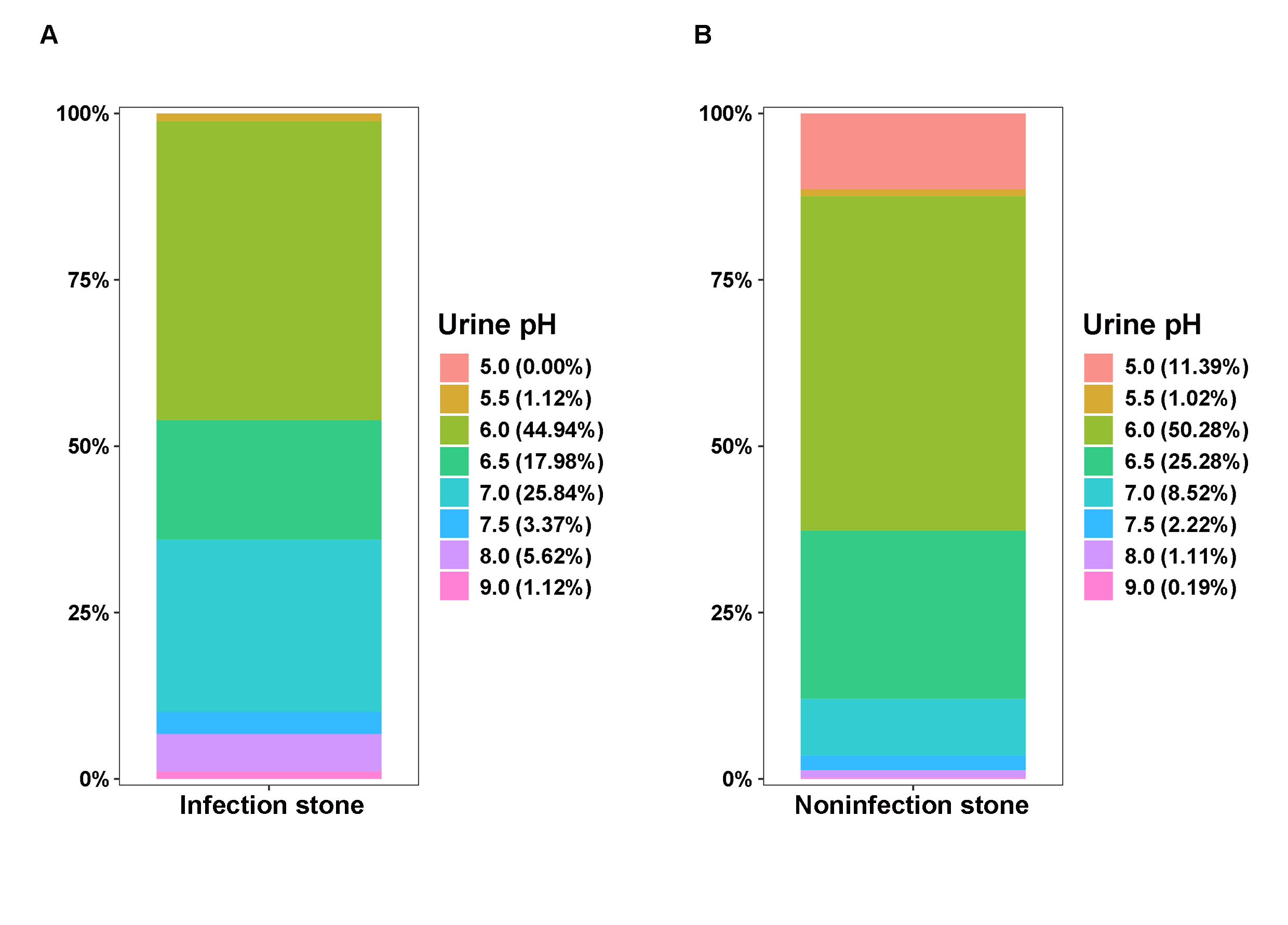

Supplement: Supplementary file 2 — Supplementary file2 (TIF 668 KB) [file 240_2023_1457_MOESM2_ESM.tif]
