# Supplementary material for: A retrospective study using machine learning to develop predictive model to identify urinary infection stones in vivo
Source: Urolithiasis. 2023 May 31;51(1):84. doi: 10.1007/s00240-023-01457-z (PMC10232574; doi:10.1007/s00240-023-01457-z)
Supplement: Supplementary file 3 — Supplementary file3 (DOCX 12 KB) [file 240_2023_1457_MOESM3_ESM.docx]

Supplementary Table S1. Summary of AUC, accuracy, sensitivity, specificity of different models in the training Set

|  | **accuracy** | **sensitivity** | **specificity** | **auc** | **95%CI** |
| --- | --- | --- | --- | --- | --- |
| SVM | 0.674 | 0.815 | 0.661 | 0.787 | (0.720, 0.854) |
| MLP | 0.689 | 0.908 | 0.671 | 0.763 | (0.694, 0.836) |
| DT | 0.779 | 0.446 | 0.807 | 0.744 | (0.674, 0.815) |
| RFC | 0.710 | 0.815 | 0.701 | 0.791 | (0.724, 0.858) |
| AdaBoost | 0.886 | 0.462 | 0.923 | 0.855 | (0.796, 0.913) |

SVM=Support Vector Machine

MLP=Multilayer perceptron

DT=Decision tree

RFC=Random Forest Classifier

AdaBoost=Adaptive boosting

AUC = area under curve
